# Using the Body When There Are No Words for Feelings: Alexithymia and Somatization in Self-Harming Adolescents

**DOI:** 10.3389/fpsyt.2020.00262

**Published:** 2020-04-08

**Authors:** Alessia Raffagnato, Caterina Angelico, Perla Valentini, Marina Miscioscia, Michela Gatta

**Affiliations:** ^1^Child and Adolescent Neuropsychiatry Unit, Department of Women’s and Children’s Health, Padua University Hospital, Padua, Italy; ^2^Department of Communication Sciences, Humanistic and International Studies: History, Culture, Languages, Literature, Arts, Media, University of Urbino ‘Carlo Bo’, Urbino, Italy; ^3^Department of Developmental Psychology and Socialisation, University of Padua, Padua, Italy

**Keywords:** somatic complaints, somatization, non-suicidal self-injury, alexithymia, adolescence, developmental psychopathology, self-harming

## Abstract

The present case-control study aimed to investigate the relationship between alexithymia and somatic complaints in the psychopathological setting of non-suicidal self-injury (NSSI). A clinical sample of 134 adolescents (115 females; 85.5%) from 12 to 18 years old engaging in NSSI was compared with a control group of 243 high-school students (157 females; 64.6%) from 13 to 18 years old. Data were collected using two questionnaires: The Youth Self-Report 11–18 and the 20 Toronto Alexithymia Scale. In both cases and controls, the presence of somatization and alexithymia was associated with more severe psychopathological problems. Analyses were conducted to explore the association between somatic disorders and alexithymia. In the clinical group, somatic complaints were reported by 95.9% of alexithymic individuals, whereas only 44.3% of alexithymic adolescents reported somatic complaints. A one-way relationship emerged between somatization and alexithymia: while alexithymia would seem to be a factor associated with self-injury, somatic disorders were strongly associated with alexithymia, but not necessarily with self-injury. Among the self-harming adolescents, those with both alexithymia and somatization had a more severe psychopathological picture than the individuals with alexithymia but no somatization. This would suggest that, in the setting of NSSI, greater difficulty with identifying feelings is associated with somatization, and alexithymia would be an attribute common to self-harming behavior and somatization, both of which are characterized by the body being used to express psychological and emotional problems. Future research could further investigate alexithymia in self-harming individuals, in relation to any presence or absence of somatic disorders, with longitudinal assessments on any differences in their manifestation of self-injury and its psychopathological correlates.

## Introduction

“Nonsuicidal self-harming, or self-injury” (NSSI) was defined by Nock and Favazza (1) as the voluntary destruction or alteration of body tissue without any suicidal intent, for purposes that are not socially approved. This category includes behavior such as self-cutting, head banging, burning, self-hitting, scratching to the point of bleeding, and interfering with the healing of a wound (1–6).

Research on NSSI has been increasing, particularly in the last two decades (7). Published empirical studies report that hospitals have been seeing rising numbers of people who engage in self-harming behavior, with or without suicidal intent, over the past 10 to 20 years (3, 8, 9). Self-harming can generally be considered a growing health problem worldwide (10), and the individuals engaging in this behavior are at high risk of subsequent suicide (4, 11), so it is important to identify them and take preventive action. While some empirical evidence shows that people tend to contact the healthcare services before a self-harming episode or suicide attempt, they often complain of physical rather than psychological problems. In a study by Idenfors et al. (12) it emerged that children and adolescents beginning to engage in self-harming behavior had more contact with healthcare services and more hospitalizations for non-psychiatric causes before any episodes of self-injury than their peers not engaging in such behavior. In a subsequent study, the same authors found that individuals who had complained of somatic disorders in the 12 months before an episode of self-injury were also at a higher risk of suicide. The authors concluded that it is important to check for a possible self-injuring or suicidal risk in adolescents who access healthcare for somatic symptoms (13). Another study by Houston et al. (14) found that two in three patients arriving at a hospital in Oxford for NSSI had been seen by a general practitioner in the month preceding the self-harming episode, and one in three had done so in the previous week. In particular, 36.1% of those who had been seen by a general practitioner within a month before the episode of NSSI had physical ailments. Approximately one in three patients had suicidal ideation during the medical examination, but only 13.3% reported such thoughts to the specialist in hospital (14).

In light of such findings, some authors have claimed that self-harming patients show their malaise through somatic rather than psychical symptoms (15). On the other hand, several studies highlighted the association between self-harming and difficulty with regulating emotions regulation, and with identifying and communicating feelings. Specifically, in adolescent age, moreover, the motives behind self-harming are mainly linked to dealing with negative emotions.

In both psychosomatic patients and self-harming individuals, the body serves as a means to cope with psychological pain. While psychosomatic patients express their psychological distress in the shape of physical disorders (e.g., headache, stomachache, nausea, sleep problems, dizziness, rashes, or other skin problems), self-harming individuals hurt the surface of their body to contain their psychological pain. Neither psychosomatic nor self-harming individuals can mentalize and express their negative feelings in words, so they use their body to show their psychological pain (16).

Empirical studies found that alexithymia, i.e., difficulty in identifying and describing feelings and modulating states of emotional arousal (17, 18), is associated with both self-harming (19–21) and psychosomatic behavior (16–18).

A limited ability to identify, understand, and express an emotional state may increase the risk of NSSI (22). An Italian study on a non-clinical adolescent population found a positive association between difficulty in identifying and describing one's feelings and NSSI behavior. It also found that dysfunctional relationships with peers and parents can raise the risk of NSSI and suicidal ideation, consequently impairing an adolescent's ability to identify their emotions (23).

In a longitudinal study conducted in New Zealand, alexithymia emerged as a predictive and proximal factor for NSSI. In the presence of mood disorders, alexithymia determined an individual's vulnerability to the use of NSSI as an avoidance coping strategy, or an alternative way to express emotions (24).

The association between alexithymia and NSSI has been analyzed in neuroimaging studies, too, using functional magnetic resonance imaging. Different patterns of activation of the supramarginal gyrus, angular gyrus, and mid-temporal gyrus emerged as a result of individuals viewing different images of facial expressions. These areas appear to be involved in identifying and understanding emotions from others' facial expressions, in empathy, and the assimilation of multisensory information. In particular, a more outward-oriented way of thinking, or a lesser tendency for introspection seem to relate to a lack of activation in response to seeing facial expressions of happiness. Functional tests on NSSI patients who reported not caring about their feelings showed lower levels of neuron activation at the sight of images of happy expressions and higher levels of activation for frightening images (25).

As mentioned earlier, difficulties with identifying and communicating feelings (i.e., alexithymia) could also be a factor common to both somatic symptoms and self-injury. In both clinical pictures, the body is used to express psychological and emotional aspects that are otherwise impossible to communicate (16). Besides, in the case of self-harming behavior in people with somatic symptoms, several authors found a strong association between their latter symptoms and signs of anxiety and depressed mood (26, 27). Bohman et al. (28) reported that adolescents with depressive disorders had more somatic symptoms (e.g., headache, abdominal pain, dizziness, nausea, tired eyes) than healthy controls. They also found that the duration and severity of their depression correlated with the number of their somatic symptoms. A strong correlation emerged between their depression, the number of somatic symptoms they experienced, and their suicidal plans, thoughts, or attempts as well. Ginsburg, Riddle, and Davies (29) made the point that children and adolescents with anxiety disorders have significantly more somatic symptoms, and their reported somatic symptoms are more severe the higher the severity of their anxiety. Studies in the literature have emphasized how depressive symptoms and anxiety coincide with NSSI (30–32), as well as with suicide (33). This gives the impression that the presence of NSSI in individuals with somatic symptoms might be mediated by comorbidity with internalizing disorders (34).

More research is needed to elucidate the relationship between somatic complaints and self-harm in young people. Some studies exploring the relationship between alexithymia and somatic symptoms (35–38)—also in clinical samples, such as patients with depression (39)—have identified an association between these two conditions, especially for difficulty in identifying feelings, however, this link is sometimes unclear or can be mediated by psychological distress (40). These features of alexithymia could represent itself, indeed, a high risk of psychological distress (41).

Parolin and colleagues (42) investigate the levels of alexithymia in young adults with substance use disorders compared to a young adolescent with psychiatric disorders and healthy individuals. In this study, the authors found higher levels of alexithymia in both clinical groups as compared to control groups, but they did not differ from each other. This study highlights that alexithymia is not associated with specific clinical disorders but is correlated with depressive symptoms.

At the same time, some authors find the strong association between psychological factors and medical condition, as fibromyalgia, psoriasis, diabetes, where psychological distress and poor quality of life correlate with higher severity of the pathology (43, 44). However, no work has been done on the combination of alexithymia and somatic symptoms with NSSI.

Hence the present study, which aimed: i) to test whether self-harming adolescents have significantly more somatic complaints (with no known medical cause) than their peers who do not engage in NSSI; and ii) to clarify the relationship between somatization and alexithymia in the setting of NSSI, and the related clinical characteristics.

## Materials and methods

### Subjects

We enrolled self-harming young people attending two neuropsychiatry units for children and adolescents in Northern Italy from January 2015 to December 2018. Participants and their parents gave their informed consent to the study (the two services have an institutionally approved protocol based on the use of standardized forms for obtaining informed consent to the administration of questionnaires and the collection of data for clinical and research purposes). Our final clinical sample consisted of 134 self-harming adolescents, 19 males (14.2% of the sample), and 115 females (85.8%), from 12 to 19 years old (mean 15.4 years, SD=1.36).

The experimental group has been enrolled with the following inclusion and exclusion criteria: a) aged between 12 and 19 years; b) presenting almost 1 episode of self-harming; c) that does not exhibit an intellectual disability [tested with the Wechsler Intelligence Scale)(45)]; d) parents signed the informed consent.

A control group was recruited from among students attending five regional high schools. The non-experimental group sees the following inclusion and exclusion criteria: a) aged between 12 and 19 years; b) have had a formal consent signed by parents (or legal ward/caregiver). After an informative interview with the headmaster, parents were sent a letter describing the research project, providing details of the test methods, and requesting that they sign a form to authorize their child's participation. The questionnaires administered to the controls were the same as those administered to the cases. They were completed anonymously in class during regular school hours, in compliance with current legislation on privacy, and the presence of an operator from our service. After ruling out respondents who admitted in the questionnaire to having taken some self-harming action, the group of controls considered in the statistical analysis amounted to 243 students: 86 males (35.4% of the sample) and 157 females (64.6%), aged from 13 to 19 years (mean 15.8, SD=1.35).

### Procedures

Patients completed the questionnaires used in the study (see list below) at the time of clinical interviews for their diagnostic assessment. During these interviews with patients, we recorded their clinical history, their reason for coming to our service, clinical aspects of their self-harming behavior.

The clinical sample was classified with certain characteristics: reason for accessing our service (patients presenting with NSSI, or with a history of such behavior; patients presenting with problems other than NSSI, who subsequently experienced NSSI episodes); frequency of self-harming episodes (occasionally or habitually, i.e., patients with fewer or more than five episodes a year, respectively); psychopathological features from administration tests' results.

### Study Protocol and Materials

Case-control study design was adopted. The cases and controls were administered two questionnaires: the 11-18 Youth Self-Report (YSR) (46); and the 20 Toronto Alexithymia Scale (TAS-20) (17). The scores obtained were first compared between cases and controls. Then comparisons were drawn between the groups into which the clinical sample was further divided.

The Achenbach questionnaires are among the scales most often used internationally for juvenile rating behavior, in the clinical setting, and research. We used the version for adolescents (YSR 11-18), which yields two profiles: one concerning competences (activities, social functioning, school performance), then how well the adolescent performs in sports, hobbies, autonomy, and socialization, and at school; the other covers behavioral and emotional problems, which can be classified as “normal,” “borderline,” or “clinical” on eight specific syndrome scales. These syndrome scales relate to various psychopathological pictures: anxiety/depression; withdrawal; somatization; social problems; thought-related problems, attention problems; aggressive and rule-breaking behavior. Problems are grouped into internalizing problems (anxiety, depression, withdrawal, somatization); externalizing problems (aggressive and rule-breaking behavior); and other problems (social problems, thought-related problems, attention problems).

The 20 Toronto Alexithymia Scale (TAS 20) is 20 items self-report questionnaire that measures the three factors defining alexithymia: “difficulty in identifying feelings,” “difficulty in communicating feelings to others,” and “externally-oriented thinking.” Respondents were classified as non-alexithymic (scores <51), borderline (scores 51–60), or alexithymic (scores >61). We used the Italian validated version of the TAS-20.

### Statistics

The statistical analyses were all conducted with the “Jamovi” statistical software. Univariate analysis of variance (ANOVA) was run to identify possible similarities or differences between the clinical and control groups as regards their psycho-behavioral profile and the alexithymia dimension. A bivariate parametric correlation analysis (Pearson's r coefficient) was conducted to investigate the relationships between clinical features and groups. Within the clinical group, analyses were run to examine the relationship between alexithymia, somatic disorders and psychopathological characteristics. The statistical tests used in this study were bidirectional, and the threshold for statistical significance was set at p < 0.05.

## Results

Frequency and percentage statistics are shown for the categorical variables corresponding to the clinical group's characteristics. We obtained information on the frequency of self-harming for 84 subjects: 30 of them (35.7%) did so occasionally, and 54 (64.3%) habitually. Information on the reasons for accessing our services was only available for 77 subjects: it was NSSI in 37 cases (48.1%); NSSI in association with attempted suicide in 4 (5.2%); affective disorders in 12 (15.6%); and problems at school in 5 (6.5%); and other reasons—including eating disorder, depression and NSSI, behavioral disorder, phobia, and social closure—in one case each (1.3%).

### Psychopathological Characteristics

ANOVA revealed statistically significant differences between the clinical group and the control group regarding emotional-behavioral problems. There were no statistically significant differences within the clinical group based on the frequency of self-injury. The descriptive statistics are shown in **Table 1**. The clinical group had more severe problems than the control group on all the scales investigated:

- *YSR 11-18*: “internalizing problems” (F=132.89, p<.001), “externalizing problems” (F=71.53, p<.001), “total problems” (F=163.73, p<.001), “social problems” (F=85.59, p<.001), “anxious/depressed” (F=85.11, p<.001), “withdrawn/depressed” (F=78.64, p<.001), “somatic complaints” (F=82.35, p<.001), and “aggressive behavior” (F= 41.73, p<.001).

**Table 1:** Average scores on the Youth Self-Report (YSR) scales for cases and controls.

CBCL	Group	M (SD)		Group	M (SD)
Anxious/depressed	Case control	70.3 (12.30) 59.1 (8.59)	Affective problems	Case control	72.3 (12.15) 56.6 (7.09)
Withdrawn depressed	Case control	69.5 (12.1) 58.5 (9.61)	Anxiety problems	Case control	64.4 (8.58) 58.5 (7.68)
Somatic complaints	Case control	63.9 (9.26) 55.6 (6.40)	Somatic problems	Case control	62.4 (9 .70) 55.1 (6.52)
Social problems	Case control	66.0 (9.68) 56.6 (8.41)	Oppositional defiant problems	Case control	58.6 (7.63) 53.8 (5.50)
Thought problems	Case control	64.6 (10.22) 54.2 (5.43)	Attention deficit/hyperactivity problems	Case control	58.1 (6.72) 53.6 (5.02)
Attention problems	Case control	63.4 (9.66) 55.4 (7.30)	Conduct problems	Case control	58 (8.70) 52.1 (4.62)
Rule-breaking behavior	Case control	59.2 (8.42) 53.9 (5.40)	Internalizing problems	Case control	69.7 (10.72) 56.1 (11.01)
Aggressive behavior	Case control	59.3 (8.53) 53.8 (5.72)	Externalizing problems	Case control	58.6 (9.66) 49.9 (8.84)
Activities	Case control	37.4 (9.39) 39.8 (8.76)	Total problems	Case control	66.2 (9.31) 53.0 (9.63)
Social	Case control	39.2 (9.74) 45.6 (8.97)			
Total competence	Case control	35.1 (9.71) 40.4 (9.27)			

To be more specific, 74% of our patients with NSSI had clinically-relevant scores for internalizing problems, as opposed to 41.2% in the control group. The situation was similar for clinically-relevant externalizing problems (identified in 28.6% of the patients and 8.2% of the controls). As for somatic problems, 22.8% of the NSSI group obtained a borderline score, and 18.1% were in the clinical range, whereas 90.5% of the control group presented no such problems. In short, our clinical group had more psychopathological issues than controls, including internalizing and externalizing problems, depression, anxiety, social difficulties, and aggressive behavior.

Pearson's correlation analysis revealed statistically significant correlations between the clinical group and all test scores (**Table 2**). Statistically significant negative correlations emerged on the Competence scales (NSSI individuals scored lower), while statistically positive correlations were seen for all the other YSR scales; that means that the clinical group has less competences and more problems. All psychopathological characteristics investigated were significantly associated with NSSI. In qualitative terms, there were stronger correlations between the clinical group and affective disorders, and specifically for thought problems in the cluster of affective problems. As for differences by gender, female sex was associated with more psychopathological problems.

**Table 2:** Correlations of the Youth Self-Report (YSR) global problems scales.

	Group	Conduct	Internalizing	Externalizing	Total problems	Somatic problems	Anxiety depression	Thought problems	Affective problems
Gender	.228**	.063	.242**	.048	.173**	.204**	.204**	.102	.202**
Group	1	.410**	.512**	.413**	.551**	.409**	.473**	.556**	.631**
Conduct		1	.359**	.777**	.598**	.301**	.349**	.532**	.473**
Internalizing			1	.513**	.879**	.622**	.855**	.687**	.829**
Externalizing				1	.799**	.416**	.481**	.601**	.567**
Total problems					1	.603**	.802**	.793**	.839**
Somatic problems						1	.482**	.530**	.550**
Anxiety depression							1	.672**	798**
Thought problems								1	.783**

**p < 0.01.

### The Alexithymia Dimension

Our assessment of alexithymia showed statistically significant differences between the clinical and control groups.

- *TAS-20*: “difﬁculties identifying feelings” (F=92.69, p<.001), “difﬁculties describing feelings” (F=35.64, p<.001), “externally-oriented thinking” (F=40.44, p<.001), “total alexithymia” (F=99.17 p<.001).

The mean score for total alexithymia in the clinical sample was M= 63.8 (SD=12.01), while in the control sample, it was M= 51 (SD=11.27). Adopting a score of 61 as a cut-off for alexithymia, the clinical group scored higher (i.e., had alexithymia), and the control group lower. In detail, 64.1% of the NSSI individuals showed clinically-relevant levels of alexithymia, while 23.4% obtained borderline scores. In the control group, 21.1% had clinical scores, and 24.8% were in the borderline range. As reported elsewhere in the literature, alexithymia is a common characteristic of self-harming individuals (**Table 3**).

**Table 3:** Average Toronto Alexithymia Scale (TAS)-20 scores for cases and controls.

TAS-20	Group	Mean (SD)
Difﬁculties identifying feelings	Case control	23.4 (6.54) 16.6 (6.16)
Difﬁculties describing feelings	Case control	17.5 (4.64) 14.4 (4.95)
Externally oriented thinking	Case control	23.0 (4.49) 20.0 (4.04)
Total alexithymia	Case control	63.8 (12.01) 51.0 (11.27)

Pearson's correlation analyses revealed statistically significant positive correlations between the clinical group and the TAS-20 sub-scales. Individuals engaging in NSSI were associated with a higher degree of alexithymia. TAS-20 factors also showed a statistically significant positive correlation with all the YSR problem scales except for social competence, with which they correlated negatively. The higher the difficulties in identifying and describing feelings, and in externally oriented thinking, the higher the scores on the psychopathological scales for internalizing and externalizing disorders (**Table 4**).

**Table 4:** Correlations between condition, gender, Youth Self-Report (YSR) scores, and Toronto Alexithymia Scale (TAS)-20 subscales.

	Difﬁculties describing feelings	Difﬁculties identifying feelings	Externally-oriented thinking	Total alexithymia
Gender	.238**	.142**	−.034	.186**
Condition	.455**	.297**	.324**	.470**
Internalizing	.630**	.591**	.347**	.701**
Externalizing	.457**	.226**	.309**	.447**
Total problems	.669**	.533**	.387**	.714**
Somatic complaints	.536**	.402**	.226**	.527**
Affective problems	.632**	.469**	.339**	.643**
Somatic problems	.453***	.359**	.224**	.463**

**p < 0.01.

### Relationship Between Alexithymia and Somatic Disorders in the Clinical Group

To investigate the link between alexithymia, somatic disorders and psychopathological characteristics, we ran analyses on the clinical group and compared it with the control group. Subjects were subgrouped by the presence of alexithymia and the presence of somatic disorders. Analysis of variance in the clinical group revealed statistically significant differences within these subgroups: NSSI patients with alexithymia had more severe emotional-behavioral problems. The same pattern emerged when these subjects were grouped by the presence or absence of somatic disorders. Alexithymia and somatization were both associated with more severe psychopathological issues. The analyses conducted on the control group produced the same results. To explore possible associations between alexithymia and somatization, a statistical analysis was carried out with the *x*^2^ test. We obtained statistically significant results in both the NSSI group (*x*^2^=5.24; p=0.02), and the control group (*x*^2^=10.7; p=0.001); see **Table 5**.

**Table 5:** Relationship between alexithymia and somatic disorders.

Somatization	Alexithymia			
	Absent		Present	
	Case	Control	Case	Control
Absent	13 (10.7%)	126 (52.1%)	59 (48.8%)	93 (38.4%)
Present	2 (1.7%)	5 (2.1%)	47 (38.8%)	18 (7.4%)
Total	15 (12.4%)	131 (54.1%)	106 (87.6%)	111 (45.9%)

The presence of somatic disorders is strongly associated with the alexithymia dimension. Only two individuals in the NSSI group had somatic disorders but no alexithymia, while 95.9% of those reporting clinically-relevant somatic complaints had alexithymia. That said, alexithymia was not necessarily associated with somatic disorders: 55.7% of the NSSI subjects with alexithymia did not report any clinically-relevant somatic complaints. Within the NSSI group, 38.8% had somatic complaints concomitantly with alexithymia. When the same analyses were run on the control group, the same one-way relationship between somatic disorders and alexithymia came to light: 78.3% of the individuals presenting somatic disorders had clinical alexithymia. The controls differed from the group of NSSI patients in that alexithymia was less strongly associated with somatization: only 16.2% of the controls had clinical levels of alexithymia concomitantly with somatic complaints. No statistically significant differences were found when we checked for possible associations between frequency of NSSI, alexithymia and somatic disorders.

Since alexithymia was not necessarily associated with the presence of somatic disorders in the group with NSSI, we run an ANOVA on this group to seek any differences between the alexithymic cases based on the presence or absence of concomitant somatization. The results showed statistically significant differences between these two subgroups.

- *YSR 11-18*: “internalizing problems” (F=18.46, p<.001), “externalizing problems” (F=5.54, p=0.021), “total problems” (F=19.34, p<.001), “anxious/depressed” (F=6.13, p=0.016), “withdrawn depressed” (F=4.01, p=0.04), “social problems” (F=5.30, p=0.023), “thought problem” (F=7.34, p=0.008), “aggressive behavior” (F=5.22, p=0.025), “affective problems” (F=12.26, p<.001), “oppositional defiant problems” (F=5.46, p=0.022)
- *TAS-20:* “difﬁculties identifying feelings” (F=8.98, p=0.003), “difﬁculties describing feelings” (F=5.89, p=0.017), “total alexithymia” (F=10.23, p=0.002)

The association between alexithymia and somatization correlated with a clinically more severe psychopathological condition. This association was more common in the group of NSSI patients than in the control group.

We conducted further analyses to clarify what distinguishes a self-harming individual among those revealing a combination of alexithymia and somatization. We considered only the subgroups with clinically-relevant levels of both alexithymia and somatization within each of the two groups (cases and controls). The results of our ANOVA showed statistically significant differences between them.

- *YSR 11-18*: “internalizing problems” (F=9.23, p=0.005), “externalizing problems” (F=13.81, p<.001), “total problems” (F=20.36, p<.001), “anxious/depressed” (F=9.70, p=0.003), “withdrawn depressed” (F=8.83, p=0.006), “social problems” (F=10.52, p=0.003), “thought problem” (F=26.33, p=<.001), “attention problems” (F=10.73, p=0.002), “rule-breaking behavior” (F=8.73, p=0.005), “aggressive behavior” (F=14.58, p<.001), “affective problems” (F=36.27, p<.001), “anxiety problems” (F=7.34, p=0.01), “oppositional defiant problems” (F=7.56, p=0.009), “attention deficit/hyperactivity problems” (F=11.01, p=0.002), “conduct problems” (F=14.37, p<.001).
- *TAS-20* “difﬁculties identifying feelings” (F=10.43, p=0.003), “externally oriented thinking” (F=6.32, p=0.016), “total alexithymia” (F=11.23, p=0.002).

The subgroup of NSSI cases with both alexithymia and somatization differed from the controls with these two concomitant conditions in that the former had more severe degrees of psychopathology, and higher difficulties with identifying feelings, and outward-oriented thinking.

## Discussion

As in the literature, which points to females engaging in self-injury more than males, both in clinical samples (47, 48), and in the general population (19, 48–51), our clinical group consisted mainly of girls, with a male to female ratio of one to six.

This study aimed to investigate the relationship between psychopathological traits, alexithymia, and somatic disorders in a sample of self-harming adolescents. More than half of our sample (64.3%) engaged habitually in self-injury (more than five episodes a year). This is a higher proportion than the figures reported in non-clinical populations (52), but similar to the rate seen in clinical populations considered in other Italian studies (20), and lower than the percentage recorded by Washburn (53) in a clinical population of adolescent inpatients and outpatients.

Regarding the reasons why our NSSI patients came to our neuropsychiatric service, about half of the sample mentioned not NSSI, but affective disorders, problems at school, phobias, social problems, behavioral issues, or eating disorders. Such disorders are often associated with self-harming behavior, and the latter might be investigated as part of the clinical assessment of patients presenting with these kinds of psychopathological issues. Much the same picture emerged in other clinical studies (19). We can also assume that most adolescents revealing self-harming behavior tend to underestimate it, and even their parents—when they are aware of it—may minimize the problem (54). Parents are frequently unaware of their children's self-harming behavior. However, a meta-analysis recently revealed that only 49% of adolescents engaging in NSSI ask for help, and only 25% of them talk to members of their family about the problem (55). General practitioners or pediatricians (who can request a neuropsychiatric examination) might have difficulty in diagnosing NSSI, especially when it is not associated with behavioral or affective disorders or suicidal behavior. A recent review found that about one in two pediatricians did not feel able to deal with a patient engaging in self-harming, and less than one in three routinely assessed their patients for NSSI (6).

The results of the present study generally show more severe psychopathological traits in the clinical population of adolescents engaging in NSSI. This confirms other clinical reports (3, 48, 51, 56–61) of several psychopathological disorders being related to self-injury, an aspect that underscores the nosographic transversality of NSSI. We hypotheses that self-harming adolescents could have significantly more somatic complaints (with no known medical cause) than their peers who do not engage in NSSI. There was a statistically significant correlation in our sample with all the scales in the YSR questionnaire, including somatic complaints. The same picture emerged in other studies using the same instrument (YSR): in a case-control study, Laukkanen et al. (62) found more pathological scores in self-injuring adolescents than controls on all the syndrome scales considered (anxiety and depression, depression and social withdrawal, somatic complaints, social problems, thought problems, attention problems, rule-breaking and aggressive behavior). Baetens et al. (63) found their NSSI group scored higher on the following DSM-oriented scales: affective problems, anxiety problems, somatic problems, attention and hyperactivity problems, oppositional-defiant problems, and conduct problems. The same differences emerged in another Italian case-control study too (20), with self-harming patients showing more severe psychopathological issues than controls in all subscales of the YSR.

It is important to emphasize, however, that internalizing disorders were qualitatively the most represented in our clinical sample, and stronger correlations were apparent between the NSSI group and the presence of affective disorders. This finding seems to be in line with other studies (19, 51, 64, 65). Some authors have concluded that the presence of a mood disorder can be a predictor of NSSI (65), as seen in other works on non-clinical adolescent populations (24, 66, 67). One hypothesis is that adolescents with mood disorders find comfort in the act of self-injury (68), as a strategy for coping with their depressive symptoms, like brooding (or rumination), to escape negative emotional and cognitive states (69–71). Among the mood disorders, bipolar disorder is characterized by strong emotional lability, and patients may be more vulnerable to acts of NSSI (72). Young people with severe depressive symptoms may use NSSI to obtain positive emotions, and as behavior that gives them social reinforcement (73).

The literature shows that the female gender is associated with more significant psychopathological problems (74). Studies on clinical samples and general populations report to a higher prevalence of females with a history of NSSI (47, 48, 50, 51). Some authors even consider female sex a risk factor for NSSI (75, 76), as well as for a range of feelings of distress, such as anxiety and depression (51, 77). Compared to their peers of the opposite sex, females tend to have a higher prevalence of psychopathological issues, especially internalizing problems, and they are associated with a higher risk of developing more severe forms of self-injury, such as suicidal behavior (78, 79).

Alexithymia appears to be a specific feature associated with self-harming behavior. In fact, a significant correlation emerged between our NSSI group and all subscales of the TAS-20 questionnaire. The risk of NSSI developing in alexithymic subjects was highlighted in a study by Norman and Borril, too (22). These findings also confirm a previous report from Gatta et al. (19) on a non-clinical population of adolescents: those engaging in self-harming had considerably more difficulty in recognizing and expressing their own and others' emotional states, suggesting a fundamental role for alexithymia in the onset of NSSI (19). Similar evidence comes from other research in Italy and New Zealand (23, 24), suggesting that alexithymia is a risk factor for NSSI as a dysfunctional coping mode, as well as by neuroimaging studies using functional magnetic resonance imaging (25).

In our results, the correlation between the YSR and TAS-20 scales goes to show how a higher difficulty with identifying and communicating feelings, and with outward-oriented thinking is associated with more serious mental issues. It is also important to emphasize that, inasmuch as a self-report questionnaire can measure psychopathological issues, the presence of alexithymic traits could just impact this.

This study aimed to clarify the relationship between somatization and alexithymia in the setting of NSSI and the related clinical characteristics. To investigate the relationship between alexithymia, somatization, and emotional-behavioral psychopathological issues, in the NSSI group, we divided the sample by the presence or absence of somatic complaints and alexithymia. Our results showed a one-way relationship between the presence of somatic disorders and alexithymia: while 95.9% of the adolescents reporting clinically-relevant somatic complaints had alexithymia, 55.7% of those with alexithymia reported no somatic complaints. There was a statistically significant association between alexithymia and somatization in the control group too, consistently with the literature (35, 36, 39), but this association was weaker in qualitative terms.

Difficulties in identifying and communicating feelings thus seem to be a factor common to both somatization and self-harming. In both cases, the body is used to express psychological and emotional issues that an individual finds impossible to communicate verbally (16).

Further analyses conducted on our sample of clinical cases aimed to look for differences between individuals with alexithymia based on whether or not they had concomitant somatization. Our results showed more severe psychopathological issues in the adolescents in our NSSI group who had clinical levels of alexithymia and somatization than in those with clinically-relevant alexithymia alone. Although we cannot identify a cause-effect relationship, we can say that concomitant somatization in self-harming individuals with clinical levels of alexithymia is associated with a more severe psychopathological picture. We, therefore, surmise that alexithymic individuals' expressions of psycho-emotional distress in the form of somatic complaints constitute a risk factor for their engaging in self-harming.

The concomitant presence of alexithymia and somatization was seen mainly in our self-harming group, and to a lesser degree in the control group. We sought to identify what distinguished the individuals who engaged in self-harming, after accounting for the association between somatization and alexithymia, in our sample of adolescents as a whole. To answer this research question, we analyzed only the individuals with both alexithymia and somatic complaints, divided by the presence or absence of NSSI. We found that the adolescents who engaged in self-harming had higher scores for psychopathological problems, and for difficulties in identifying feelings, and in outward-oriented thinking.

## Conclusion

This study aimed to explore the relationship between alexithymia, somatic disorders, and psychopathological traits in a group of self-harming adolescents, as compared with a control group. Our results indicated that individuals who engaged in NSSI had more problems in the internalizing and externalizing domains than the control group. There was evidence of the nosographic transversality of self-injury. In qualitative terms, internalizing disorders (and affective disorders in particular) were the factor that most strongly characterized the clinical group. This finding has important implications for clinical practice, and for primary and secondary prevention.

Self-harming individuals had more severe somatic disorders and alexithymia. Our results showed a one-way relationship between somatization and alexithymia (with somatic complaints reported by 95.9% of our alexithymic adolescents, while only 44.3% of alexithymic adolescents reported somatic complaints). Alexithymia would thus seem to be a factor associated with self-injury, whereas somatization would be closely associated with alexithymia, but not necessarily with self-injury. It may be that an alexithymic trait is like a common denominator in the use of the body instead of words to express emotion in the case of psychological disease: through somatization when the psychic conflict is displaced inside the body; or through NSSI when the emotional dysregulation and negative feelings prevail.

In our NSSI sample, the combination of alexithymia with somatization was associated with a more severe psychological picture than alexithymia alone. It could be that, in the context of NSSI, higher difficulty with identifying is associated with somatization. This leads us to suggest, once again, that alexithymia is an attribute shared by self-harming and somatization, both of which are characterized by the use of the body to express an emotional problem. Somatic complaints also appeared to be associated with more severe psychopathological issues in our sample, however, so it may be that the former was a manifestation of the latter.

Further longitudinal research is needed on alexithymia in NSSI, vis-à-vis the presence or absence of associated somatic disorders, to identify any differences in the manifestation of self-injury and its psychopathological correlates. Moreover, further researches should consider causal relationships. As regards methodology, the main limitation of the present study lies in our use of self-report tools and the lack of any clinical assessment. Since alexithymia can hinder individuals' proper identification and description of feelings, future research could include multiple informants, also considering the clinician's opinion.

## Data Availability Statement

The datasets analyzed for this study will be made available by the authors, without undue reservation, to any qualified researcher.

## Ethics Statement

The studies involving human participants were reviewed and approved by “Comitato Etico per la Ricerca Clinica” Aulss 16 Euganea. Written informed consent to participate in this study was provided by the participants' legal guardian/next of kin.

## Author Contributions

AR: wrote the first draft of this paper, literature review, and discussion of results. CA: wrote the first draft of this paper, data analysis, and discussion of results. PV: database and discussion of results. MM: discussion of results, and wrote revisions. MG: study design, discussion of results, and conclusion.

## Conflict of Interest

The authors declare that the research was conducted in the absence of any commercial or financial relationships that could be construed as a potential conflict of interest.
